# Prevalence of Visual Impairment in Preschool Children in Southern China

**DOI:** 10.3389/fpubh.2022.755407

**Published:** 2022-04-04

**Authors:** Hongxi Wang, Kunliang Qiu, Shengjie Yin, Yali Du, Binyao Chen, Jiao Jiang, Dandan Deng, Mingzhi Zhang

**Affiliations:** Joint Shantou International Eye Center of Shantou University and the Chinese University of Hong Kong, Shantou, China

**Keywords:** preschool children, vision screening, spectacles, interocular difference, visual impairment

## Abstract

**Purpose:**

The goal of this study is to assess the prevalence and distribution of visual impairment in preschool children in southern China.

**Methods:**

Preschool children aged 36–83 months were enrolled in a vision screening program in Shantou City. Visual acuity test and non-cycloplegic refraction were conducted. According to the American Academy of Ophthalmology (AAO) guidelines, visual impairment was defined as uncorrected visual acuity (UCVA) in either eye <20/50, 20/40, and 20/32 in children aged 36–47, 48–59, and 60–83 months, respectively, as well as an interocular difference (IOD) of ≥ two lines of UCVA.

**Results:**

The UCVA test was successfully performed on 7,880 children (94.6% of the enrolled population). A total of 938 (11.9%; 95% CI 11.2–12.6) children were found to have reduced UCVA in the worse eye, and 393 (5%; 95% CI 4.5–5.5) of the children had an IOD of two or more lines. Combining the reduced UCVA with the IOD criteria identified 1,032 (13.1%; 95% CI 12.4–13.8) children with visual impairment. UCVA in preschool children improves with age naturally and boys have slightly better age-adjusted UCVA than girls. Causes of reduced visual acuity included uncorrected refractive error, amblyopia, congenital cataract, and others. The cylindrical diopter in the right eye of children with reduced vison was higher than that of children with normal vision (1.19 ± 1.05 vs. 0.52 ± 0.49, *P* < 0.001). A total of 146 (1.9%, 95% CI 1.6–2.2) of the preschool children wore spectacles. The proportion of wearing spectacles increased with age (χ^2^ = 35.714, *P* < 0.001), but with IOD increasing by.1 logMAR, the odds of wearing spectacles decreased by 44.8%.

**Conclusion:**

This study provided data on the prevalence of visual impairment in preschool children in China by large-scale school-based vision screening. Further studies should be conducted to verify the benefit from vision screening.

## Introduction

Childhood visual impairment and blindness are major public health issues in the world, and are also one of the priorities in disease control of the global initiative for the elimination of avoidable blindness (1, 2). Preschool stage is a special period in which the physiology and anatomy of the visual system are malleable, and visual deprivation during this critical period can result in permanent visual loss that cannot be fixed by any corrective means (3, 4). On the other hand, even if one is not amblyogenic, visual problems at preschool age, such as uncorrected refractive error, can interfere with daily life and schoolwork (5). Therefore, as recommended by many healthcare specialists and governments, vision screening for preschool children is an integral part of preventive pediatric healthcare, which has utility for identification of children with vision problems and leads to interventions to improve the quality of life (6, 7).

However, representative and comparative data regarding the prevalence of visual impairment in preschool children are still scarce. Major barriers to preschool vision screening, as reported, are time-consuming screening tests and uncooperative children, along with some social barriers including lack of awareness, inconvenience, and insufficient eye care providers (6). Specialists in Taiwan investigated the accuracy of vision-screening tests and suggested that uncorrected visual acuity (UCVA) was probably the best single-instrument test for preschool vision screening in developing countries with low resources (8). Similarly, Mingguang He provided that using UCVA or interocular difference (IOD) in vision screening and referral in preschool children is pragmatic (9).

Recently, the National Health Commission (NHC) of the People's Republic of China (PRC) has published service specifications for pediatric eye care and vision examination, which has an important practical significance for preschool children vision screening. In order to evaluate the eye health of preschool children, it is essential to have updated information on the prevalence of visual impairment. Nevertheless, there were only few studies conducted on large-scale school-based vision screening for preschool children in China.

This study aimed to assess the prevalence and distribution of reduced visual acuity (VA) in preschool children in southern China in a setting of fast school-based vision screening.

## Methods

### Subjects and Sites

This was a cross-sectional school-based study conducted from 2017 to 2019 in Shantou, a city in eastern Guangdong province, southern China. The city of Shantou, with representative demographic and socioeconomic characteristics, has a relatively stable population of 5.59 million (in 2017) and an average annual disposable income per capita of 22,521 Yuan (US $3,521, ranking No. 9 among the 21 cities in Guangdong province), which was comparable to the national average level (25,974 Yuan, US $4,061 in 2017). Shantou has six districts and one county. According to statistical yearbooks from the Shantou City Bureau of Statistics (link: https://www.shantou.gov.cn/tjj/tjsj/tjnj/index.html), in 2017, there was a total of 923 kindergartens serving 185,089 children in Shantou.

With the approval and support of the Shantou City Bureau of Education and Bureau of Health, the Joint Shantou International Eye Center (JSIEC), one of the top tertiary ophthalmic hospitals in southern China, has been carrying out a long-term large-scale vision screening program for preschool children in Shantou since 2017. This study, as a part of the screening program, was mainly focused on the prevalence of visual impairment in preschoolers.

Preschool children aged 36–83 months attending selected kindergartens were enrolled. Based on the statistical parameters prevalence (20%), error expected (1%), confidence (95%), upward adjustment for cluster sampling design (25%), and non-participation (5%), we calculated a sample size requirement of 7,828. The sampling process was as follows: we first listed kindergartens and the corresponding number of preschoolers; to ensure screening efficiency, we ruled out kindergartens with <100 preschoolers; then, we performed simple random sampling to select kindergartens in the screening list one by one, and simultaneously accumulated the number of preschoolers until it reached the sample size requirement. All preschoolers in the selected kindergartens were enrolled. This study adhered to the Declaration of Helsinki and was approved by the Human Medical Ethics Committee of JSIEC.

### Vision Screening

We conducted fast vision screening tests in a sequential order: visual acuity, non-cycloplegic autorefraction (NCAR), strabismus detection, and slit-lamp examination. All the tests were carried out by trained and experienced technicians from the JSIEC.

Before the UCVA test was conducted, the preschool children were taught to be familiar with the shape of letter “E” and the definition of directions by the technicians until they passed a binocular pretest using the biggest optotype at near distance. A retro-illuminated (300 cd/m^2^) standard logarithm of the minimum angle of resolution (LogMAR) chart with tumbling-E optotypes (GB 11533-2011, a national standard published by NHC of PRC) was used for UCVA test at 5 m. The standard operating protocol of VA measurements was started at a distance of 5 m with the top line (20/200) and then continued by dropping down line by line if all the optotypes were correctly identified. If, at any level, a child failed to complete a line, the test was finished and, VA was recorded as the smallest size in which the child correctly identified at least half of the optotypes. If the top line at 5 m was missed, the child was asked to step forward until the first line was successfully completed, and then VA would be recorded as 20/1,000 multiplying the distance between the child and the chart. If no optotypes could be identified on the chart, the visual acuity was assessed as counting fingers, hand movements, light perception, or no perception of light. For each testable child, we first covered the left eye to test the right eye. Then, the same procedure was followed for the left eye. For children younger than 4 years old, a re-test was conduct to confirm the results. Children with VA difference of more than one line in any eye were treated as uncooperative. In addition, for children wearing glasses, presenting VA (using current spectacles) was also measured. Then, the technicians used handheld Spot Vision Screener (Welch Allyn, Skaneateles Falls, NY, United States) to simultaneously detect the NCAR for both eyes 3 times and obtain the average value. If the difference between any of two readings from an eye was >0.5 diopters (D), re-measurement was conducted for that eye. Cylinder values and interocular difference of spherical equivalent were used for analysis of astigmatism and anisometropia, respectively, since it has been reported that astigmatism has no significant association with cycloplegia (8), and that Spot Vision Screener was efficient in detecting anisometropia (10). Besides, corneal light reflex and cover-uncover tests, near and at a distance, to detect strabismus, as well as slit-lamp examination to examine the eyelids, cornea, and lens, were conducted.

### Referral Criteria and Definition

We used referral criteria based on a monocular distance visual acuity test and interocular difference from the Pediatric Eye Evaluations Preferred Practice Pattern guidelines 2017 (11) by the American Academy of Ophthalmology (AAO) for referral (Table 1).

**Table 1:** Age-specific criteria for referral in preschool vision screening.

**Age, month**	**Uncorrected or present visual acuity in either eye**	**Interocular difference**
36–47	<Snellen 20/50 (0.4 logMAR)	≥two lines or ≥0.2 logMAR
48–60	<Snellen 20/40 (0.3 logMAR)	≥two lines or ≥0.2 logMAR
61–83	<Snellen 20/32 (0.2 logMAR)	≥two lines or ≥0.2 logMAR

Reduced VA is defined as uncorrected or present VA in either eye worse than 20/50, 20/40, and 20/32 for children aged 36–47, 48–60, and 61–83 months, respectively. Spherical equivalent refraction (SER) is defined as the spherical diopters added to half of the cylindrical diopters (12). Refractive errors include myopia, hyperopia, astigmatism, and anisometropia. Myopia is defined as a non-cycloplegic SER < −0.5 D with reduced UCVA. Hyperopia is defined as non-cycloplegic SER >0.5 D. Astigmatism is defined as absolute NCAR cylindrical diopter >0.75 D. Anisometropia is defined as interocular difference of SE > 2 D. Amblyopia is defined as reduced corrected VA with definitive amblyopic causes.

Children requiring medical or surgical treatment beyond what could be provided on-site were referred to the JSIEC for further diagnosis and therapy. Uncooperative children were informed for retest within 6 months. If retesting could not be performed, referral for a comprehensive eye evaluation was suggested.

### Data Management and Analysis

Children with successful VA testing on both eyes were considered as the study population for analysis. Forms for vision screening were reviewed for completeness and accuracy before data entry. The children were divided based on age: 36–47 months (3-year-old), 48–59 months (4-year-old), 60–71 months (5-year-old), and 72–83 months (6-year-old). We also divided the children into 2 groups according to birth month: January to August and September to December, since in China, the latter would attend preschool in the next year.

Descriptive statistics were conducted to analyze the prevalence and distribution of reduced VA and current use of spectacles. We assessed for age and sex difference in distribution of reduced VA by chi-square tests. A linear mixed effects model (random intercept and slope) was used to investigate the association of visual acuity in both eyes with fixed factors (sex, month age, birth month, and year of examination) and random factors (kindergartens), with interocular correlation and clustering effect in kindergartens adjusted. Logistic regression was performed to analyze the influence of IOD on odds of wearing spectacles. Qualitive data were presented as ratio and 95% confidence interval (CI), and quantitative data were presented as mean ± standard deviation (SD). A statistical analysis was conducted using the SPSS software (Version 23.0). A *P* < 0.05 was considered to be significant.

## Results

### Subjects

Thirty-two kindergartens were selected as sites for vision screening, and 8,332 preschool children were enrolled (Table 2). These kindergartens were distributed in six districts or one county across Shantou, with 20 (62.5%) in urban areas and the others in rural areas. All of the selected kindergartens participated in the vision screening program. UCVA tests were successfully performed on 7,880 (94.6%; 95% CI 94.1–95.1) children. Considering the device, transport expense, and human cost, the average cost of vision screening for each child in this study was 43 Yuan (US $6.7).

**Table 2:** Demographic characteristics of the study population.

	**Enrolled children / *N***	**Examined children /*N* (%)**
No. of children	8,332	7,880 (94.6)
Boy	4,225	3,959 (93.7)
Girl	4,107	3,921 (95.5)
Age, month
36–47	769	539 (70.1)
48–59	2,188	2,019 (92.3)
60–71	3,196	3,151 (98.6)
72–83	2,179	2,171 (99.6)

The refraction of children cooperative with the VA testing was detected using Spot Vision Screener. Cooperation rate was significantly lower in boys (93.7%; 95 CI 93.0–94.4) than in girls (95.5%; 95% CI 94.8–96.1) (χ^2^ = 12.674, *P* < 0.001).

Boys constituted 50.2% (95% CI, 49.1–51.3) of the cooperative children, ranging from 44.2% (95% CI 40–48.4) in 3-year-olds to 51.5% (95% CI 49.4–53.6) in 6-year-olds. The average age of the examined children was 64.15 ± 10.12 months, with the boys, on average, 0.6 months younger than the girls (64.45 ± 9.96 vs. 63.85 ± 10.27, *P* =0.008).

### Uncorrected Visual Acuity

**Figure 1:**
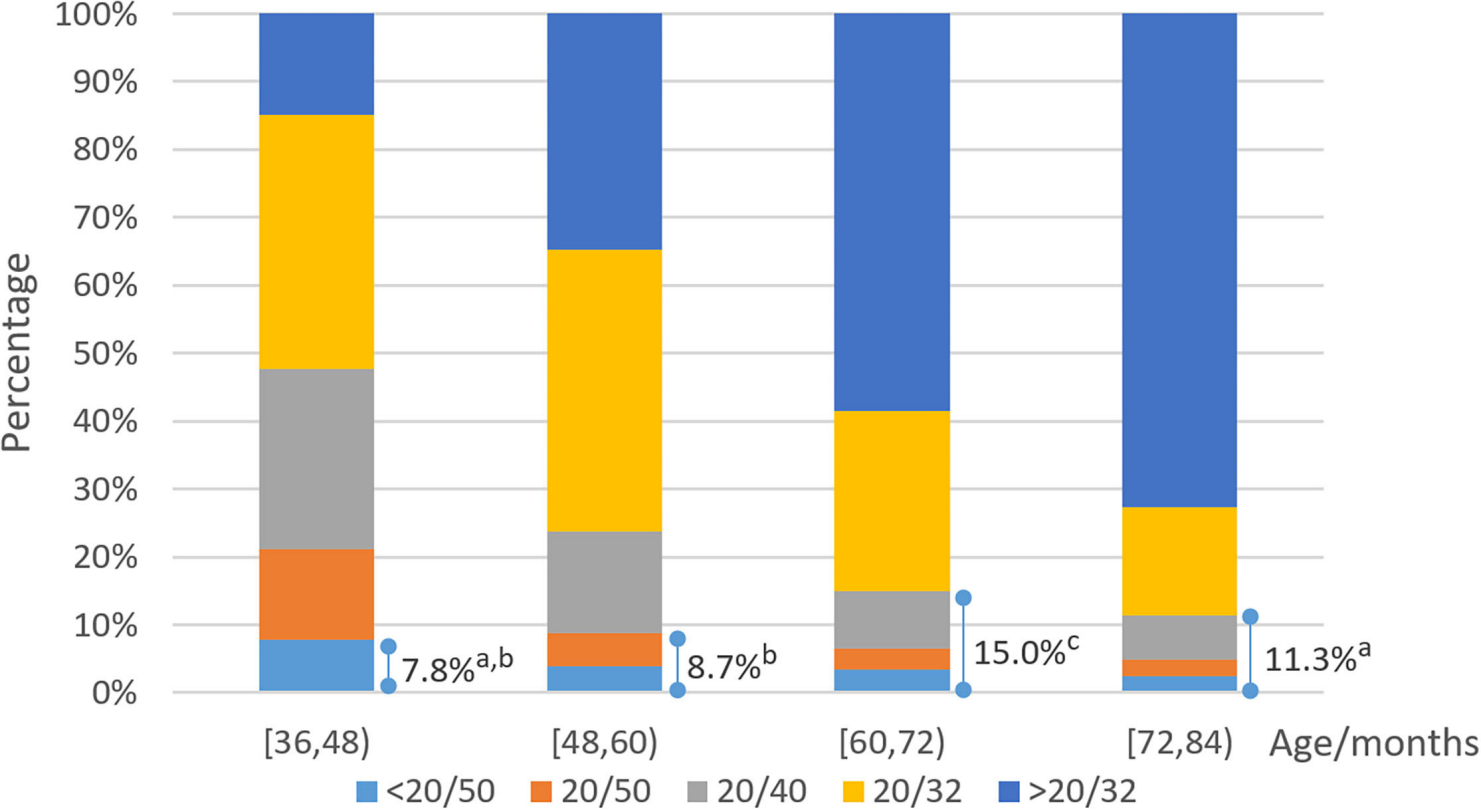
Distribution of uncorrected visual acuity (UCVA) in the worse eye. Lines and corresponding accumulating percentages denoted reduced visual acuity proportions that should be referred according to target conditions. Each subscript letter denotes a subset of age categories whose column proportions do not differ significantly from each other at the 0.05 level.

A total of 938 (11.9%; 95% CI 11.2–12.6) children were found to have reduced UCVA in the worse eye. Reduced UCVA in both eyes was found in 453 (5.7%; 95% CI, 5.2–6.3) of the children. Distributions of UCVA in the worse eye by age are shown in Figure 1. The prevalence of reduced VA differed among the age groups (χ^2^ = 58.526, *P* < 0.001). UCVA improved with age (*r* = −0.302, *P* < 0.001, Pearson correlation analysis for age in months with logMAR visual acuity in the worse eye).

**Figure 2:**
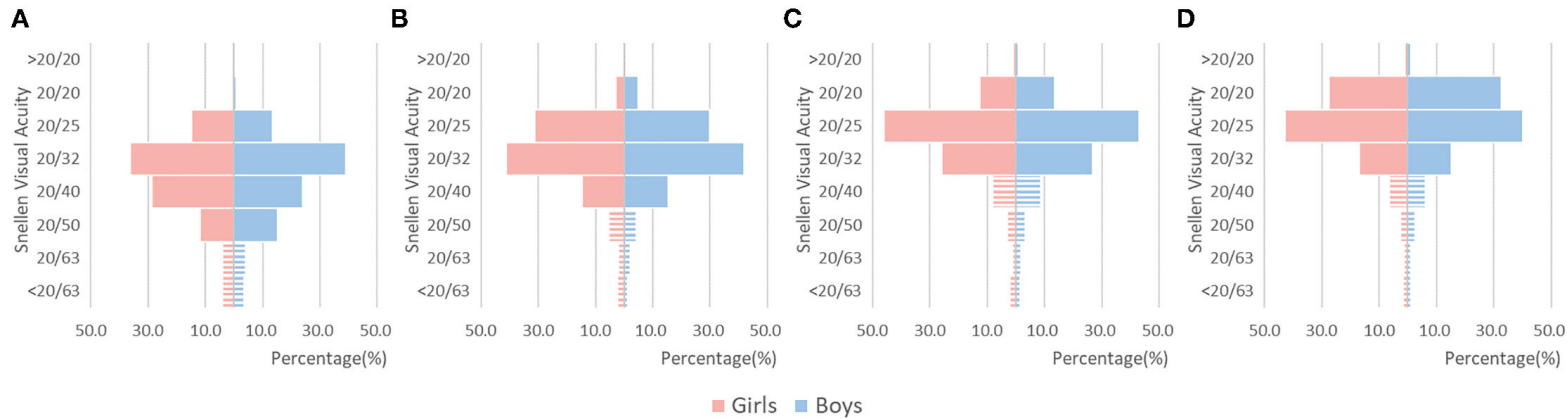
Distribution of UCVA in the worse eye by sex. Bars in strip showed reduced visual acuity proportions. **(A)** Children aged 36-47 months. **(B)** Children aged 48-59 months. **(C)** Children aged 60-71 months. **(D)** Children aged 72-83 months.

The boys had slightly better age-adjusted UCVA than the girls in the worse eye (LogMAR.161 ± 0.002 vs.0.168 ± 0.002, *P* = 0.03). Distributions of UCVA in the worse eye by sex are presented in Figure 2. There was no significant difference in the prevalence of reduced VA between the boys and the girls (*P* = 0.86, 0.16, 0.348, and 0.618, chi-square analysis for children aged 36–47, 48–59, 60–71, and 72–83 months, respectively).

A further analysis adjusting for the clustering effect on kindergartens and the interocular correlation with linear mixed effects models (Table 3) confirmed the trend or association between UCVA and factors including age and sex. In addition, UCVA was significantly correlated with year of examination, with a trend of increasing UCVA year by year (*P* < 0.001). The difference in UCVA of children born before and after September was not statistically significant (*P* = 0.795).

**Table 3:** Linear mixed effects model assessing the effect of factors on uncorrected visual acuity^a^, adjusting for clustering effect on kindergartens and interocular correlation.

**Variable**	***β*** **coefficient^b^**	***P***
Age (month)	−0.004 ± 0.0001	<0.001
Sex^c^
Boy	−0.005 ± 0.003	0.039
Birth month^d^
January to August	−0.001 ± 0.003	0.795
Year of examination^e^
2019	−0.035 ± 0.004	<0.001
2018	−0.012 ± 0.003	<0.001

*a. Uncorrected visual acuity (UCVA) recorded in logarithm of minimal angle of resolution (LogMAR). b. β coefficients of variables in the linear mixed effects model were presented as standard error. c. The variable “Girl” was set to 0. d. The variable “September to December” was set to 0. e. The variable “2017” was set to 0*.

### Interocular Difference of Uncorrected Visual Acuity

There were 393 (5%; 95% CI 4.5–5.5) children with an IOD of two or more lines. Most of the children showed an IOD of less than two lines at all ages (Table 4), without significant sex differences (*P* = 0.255). The prevalence of IOD of two or more lines ranged from 6.7% in children aged 3 to 4.5% in children aged 6.

**Table 4:** Distribution of UCVA interocular difference (IOD) in the reference population.

	***n***	**UCVA IOD, lines and logMAR equivalent**			
		**0, 0.0**	**1, 0.1**	**2, 0.2**	**>2, >0.2**
Age, month					
36–47	539	312 (57.9)	191 (35.4)	29 (5.4)	7 (1.3)
48–59	2,019	1,275 (63.2)	637 (31.6)	80 (4.0)	27 (1.3)
60–71	3,151	2,064 (65.5)	933 (29.6)	107 (3.4)	47 (1.5)
72–83	2,171	1,443 (66.5)	632 (29.1)	71 (3.3)	25 (1.2)
Sex					
Boys	3,959	2,522 (63.7)	1,233 (31.1)	155 (3.9)	49 (1.2)
Girls	3,921	2,572 (65.6)	1,160 (29.6)	132 (3.4)	57 (1.5)

*Data presented in the form of N (%)*.

Combining the reduced UCVA in the worse eye with significant IOD increased the number of referred children to 1,032 (13.1%; 95% CI 12.4–13.8), with 94 more when only the criteria for UCVA were used.

### Current Use of Spectacles

In total, 146 (1.9%, 95% CI 1.6–2.2) of the preschool children wore spectacles, and there was a trend of increased proportion of wearing spectacles with age (χ^2^ = 35.714, *P* < 0.001). Among the children wearing spectacles, there were 113 with binocular reduced UCVA and 23 with monocular reduced UCVA. Sixteen of the children wearing spectacles had a UCVA of not less than 20/40 but without amblyopic risks. Distributions of the present VA by age are shown in Table 5. There were 53 children with reduced present VA in at least one eye and 27 children with bilateral reduced present VA.

**Table 5:** Distribution of present visual acuity of the children wearing spectacles.

**Present visual acuity**	**Worse eye**	**Better eye**
36–47 m	3	
≥0.4	3	3
<0.4–0.3	0	0
<0.3	0	0
48–59 m	15	
≥0.5	8	12
<0.5–0.3	5	3
<0.3	2	0
60–71 m	62	
≥0.6	37	48
<0.6–0.3	21	13
<0.3	4	1
72–83 m	66	
≥0.6	45	56
<0.6–0.3	18	9
<0.3	3	1

Among the 393 children with significant IOD, 46 (11.7%; 95% CI 8.5–14.9) wore spectacles. After controlling for the effect of sex, age, and UCVA in the worse eye by binary logistic regression, the association between wearing spectacles or not and IOD was shown to have a statistical significance: with IOD increasing by 0.1 logMAR, the odds of wearing spectacles decreased by 44.8% (*P* = 0.003).

### Causes of Reduced Visual Acuity and Other Ocular Anomalies

Among the 938 children with reduced VA in the worse eye, astigmatism of at least 0.75 D in NCAR cylinder was found in 71.4% of right eye and in 74.9% of left eye, and in 82.3% of either eye. Astigmatism of 2 D or greater in either eye was found in 372 (39.7%) of the children. The astigmatism in children with reduced vison in the right eye was significantly severer than that in children with normal vision (1.19 ± 1.05 vs. 0.52 ± 0.49, *P* < 0.001). The mean non-cycloplegic spherical equivalent in the right eye of children with reduced vision was −0.16 ± 1.68 D. Other findings included 63 with anisometropia 2 D or more, 3 with ptosis of at least one eye, 3 with corneal opacity, 1 with corneal foreign body, 1 with postoperative corneal suture, 2 with intraocular lens, 4 with congenital cataract, and 26 with manifest strabismus.

## Discussion

This study conducted a school-based vision screening in southern China on about 8,000 enrolled preschool children. Using the UCVA and IOD criteria, we identified that 13.1% of the preschool children required referral for further ophthalmic evaluation.

The cost-benefit analysis revealed that the cost of vision screening for each child was lower in the current large-scale school-based screening compared with a previous study (13, 14). In practice, preschool vision screening is thought as an intractable issue. Besides the low cooperation degree with a series of time-consuming screening tests, another significant challenge is that the screening test protocols and results may vary among different countries or areas (5–7, 15). The UCVA test is less time-consuming and easier to master for screening technicians, and has high value in large-scale population-based screening, especially in areas with insufficient vision-screening technicians (8, 9). Most of the preschool children in the study were testable and cooperative with the tumbling-E chart, comparable with other studies (9, 15, 16).

However, a difference in the prevalence of visual impairment between this report (13.1%) and previous studies [42.8% for 3–6-year-old children in Shenzhen (9), 4.1% for 6-year-old children in Australia (17), 7% for 2–7-year-old children in Taiwan (8)] should be noted, which could be partly attributed to methodology. For instance, most of the previous studies reported and set the UCVA cutoff of visual impairment based on the Early Treatment Diabetic Retinopathy Tumbling E chart (5 optotypes in a line) or HOTV test, while GB 11533-2011 Tumbling E chart was used in this study.

Generally, the adopted UCVA cutoffs were based on clinical evidence on the relationship between UCVA and refraction. However, selecting proper UCVA cutoffs for preschool children is not straightforward, and different criteria were used across different studies (9). We selected the conservative AAO guidelines to define the target condition, which could reduce unnecessary referral, although a group of children with hyperopia and astigmatism who have unaffected vision might be excluded.

This study provided evidence that UCVA in preschool children improved with age naturally. As shown in the mixed effects model analysis, the annual cumulative change in UCVA was −0.048 logMAR, less than the change in the AAO referral criteria, which partly explained the increasing prevalence of reduced VA from age 3 to age 5. The AAO criteria identify many children with reduced UCVA at age 5. Nevertheless, the relatively lower prevalence of reduced UCVA at age 6 (using the same cutoff for age 5) suggested that most of the children have increased UCVA within 1 year without any need for intervention. A large longitudinal survey revealed that 69.8% of children with a visual acuity of 20/32 in the worse eyes at age 7 achieved a 20/20 acuity by age 16 (18). Even so, longitudinal studies should be conducted to confirm the natural improvement of VA with age in preschoolers.

The sex difference in visual acuity varied from different studies. We found that boys had slightly better UCVA than girls, which is in accordance with findings from some studies (12, 17, 19) but also in contrast with those from others (20). The causes of visual impairment included uncorrected refractive error (myopia, high hyperopia, astigmatism, and anisometropia), amblyopia, strabismus, congenital cataract, and others. In children with reduced vision, astigmatism was severer than that in children with normal vision.

It was reported that the prevalence of myopia was significantly higher in higher grades for children of same age (21). However, our findings revealed no significant difference in UCVA in preschool children born before and after September. This could be attributed to very low prevalence of myopia among preschoolers (17) and less near work in kindergarten. In addition, we used age in months as a variable in the mixed effects model analysis, which accounted for much more variance than when age in years was used. Further studies based on cycloplegic refraction should be conducted to confirm the relationship between refractive change and entrance age in preschoolers.

Purposes of wearing glasses included refractive correction, amblyopia therapy, and controlling ocular alignment (22). It was recommended that prescript of spectacles for preschool children could be postponed if without amblyogenic risks, even if UCVA is less than Snellen 20/40, since the visual demands were low (23). Our study revealed that the proportion of wearing spectacles decreased with IOD. This could be reasonable, because for children with high IOD, the UCVA in the better eye might meet the need for daily life, so, they might not complain about blurred vision. Therefore, the parents were not aware of the issue and ignored the eye health assessment and intervention. However, significant IOD of UCVA was evidence of binocular imbalance and a potential hint for monocular amblyopic risk. If with normal vision in the better eye, the reduced UCVA in the worse eye would be easily ignored, especially for 3-year-old children. What is worse, even if they were informed by ophthalmologists about risks of significant IOD, most parents still indicated that wearing glasses might be harmful to the eyes and should be delayed in children (24).

Based on what were found in this study, we recommended that routine vision screening for preschool children should be conducted. Kindergarten-based annual screening, including uncorrected and present visual acuity tests, non-cycloplegic autorefraction (NCAR), strabismus detection and slit-lamp examination, is feasible and cost-effective for detecting children who require referral for further evaluation and therapy. Besides, propaganda and education should be conducted for parents and kindergarteners to facilitate the recognition of preschoolers' visual problems.

Limitations of this study should be taken into consideration. First, we did not conduct cycloplegic refraction, which is regarded as the gold standard in previous studies focusing on the verification of vision screening tests. Therefore, the SE refraction was more myopic in preschool-aged children. Nevertheless, this time-consuming test was not suitable for large-scale screening, and our vision screening tests were fast and simple. Second, the selected kindergartens were derived from a screening list rather than statistical sampling, which reduced the representativity. Since this study was a part of a long-term vision screening program, further analyses for more representative and comprehensive reports would be conducted.

Notwithstanding its limitations, this study had several strengths, including relatively large sample size, as well as well-organization and arrangement of school-based screening with the support of the government.

In conclusion, we provided school-based data on the prevalence of reduced VA in preschool children in China with a large sample. A significant portion of preschool children with visual impairments, such as uncorrected refractive error and amblyopia, required referral for ophthalmic evaluation. Further studies should be conducted to verify the program's sustainability and benefit from vision screening.

## Data Availability Statement

The raw data supporting the conclusions of this article will be made available by the authors, without undue reservation.

## Ethics Statement

The studies involving human participants were reviewed and approved by Human Medical Ethics Committee of Joint Shantou International Eye Center. Written informed consent to participate in this study was provided by the participants' legal guardian/next of kin.

## Author Contributions

HW and MZ designed this study. DD, YD, BC, SY, and HW collected and measured data. HW and JJ analyzed data. HW, KQ, and MZ prepared the first draft and finalized the manuscript based on comments from all other authors. All authors discussed the results and commented on the manuscript. All authors contributed to the article and approved the submitted version.

## Funding

This study was supported by Guangdong Science and Technology Special Fund Project (210717146900448), Science and Technology Innovation Strategy Special Fund Project of Guangdong Province ([2018]157-46), and Intramural Grant of Joint Shantou International Eye Center (20-007 and 20-036).

## Conflict of Interest

The authors declare that the research was conducted in the absence of any commercial or financial relationships that could be construed as a potential conflict of interest.

## Publisher's Note

All claims expressed in this article are solely those of the authors and do not necessarily represent those of their affiliated organizations, or those of the publisher, the editors and the reviewers. Any product that may be evaluated in this article, or claim that may be made by its manufacturer, is not guaranteed or endorsed by the publisher.
